# Increased Tuberculosis Incidence Due to Immunotherapy Based on PD-1 and PD-L1 Blockade: A Systematic Review and Meta-Analysis

**DOI:** 10.3389/fimmu.2022.727220

**Published:** 2022-05-19

**Authors:** Kewei Liu, Dongpo Wang, Cong Yao, Min Qiao, Qing Li, Weicong Ren, Shanshan Li, Mengqiu Gao, Yu Pang

**Affiliations:** ^1^Department of Bacteriology and Immunology, Beijing Chest Hospital, Capital Medical University/Beijing Tuberculosis & Thoracic Tumor Research Institute, Beijing, China; ^2^Department of Tuberculosis, Beijing Chest Hospital, Capital Medical University/Beijing Tuberculosis & Thoracic Tumor Research Institute, Beijing, China; ^3^Department of Radiology, Beijing Chest Hospital, Capital Medical University/Beijing Tuberculosis & Thoracic Tumor Research Institute, Beijing, China

**Keywords:** PD-1, immunotherapy, tuberculosis, mortality, meta-analysis

## Abstract

**Objectives:**

In this study, we conducted a systematic review to determine tuberculosis (TB) incidence due to immunotherapy with programmed cell death protein-1 (PD-1)/PD ligand (PD-L1) blockade in cancer patients.

**Methods:**

We searched PubMed, Cochrance Library, Excerpt Medica Database (Embase), ClinicalTrials.gov, Chinese BioMedical Literature Database (CBM), China National Knowledge Infrastructure Database (CNKI), Wanfang and China Science and Technology Journal Database to identify studies between January 1, 2000 and April 30, 2021, on the reports of TB cases in patients treated with PD-1/PD-L1 blockade. Methodological quality of eligible studies was assessed, and random-effect model meta-analysis was performed to generate the pooled incidence estimate of TB cases in patients undergoing PD-1/PD-L1 therapy.

**Results:**

We initially identified 745 records, of which 27 studies ultimately met the inclusion criteria and were included in our meta-analysis. A total of 35 TB cases occurred among patients treated with PD-1/PD-L1 blockade. Nivolumab (51.4%) was the most frequently used PD-1/PD-L1 blockade for cancer treatment. In addition, pulmonary TB was the most common form of tuberculosis seen in 77.1% cases. Clinical outcomes were recorded in 18 patients, of whom 77.8% were cured or achieved remission, and 22.2% were died of TB. Pooled analysis determined that the TB rate in this population was 2,000 cases per 100,000 persons, and the estimated rate for TB associated with PD-1/PD-L1 blockade was 35 times higher than that in the general population.

**Conclusion:**

To conclude, our results demonstrate that the clinical use of PD-1/PD-L1 inhibitors significantly increases risk of TB reactivation. An extremely high mortality rate due to TB disease is noted in the patients with PD-1/PD-L1 blockade.

## Introduction

Immune-checkpoint inhibitors (ICIs) that target cytotoxic T-lymphocyte antigen-4 (CTLA-4) and programmed cell death protein-1 (PD-1) comprise the most remarkable cancer therapy advancement over the past decade (1). In fact, ICIs targeting the PD-1 pathway, a key mediator of local immunosuppression within the tumour microenvironment, have already been used to block cancer progression in clinical practice (2). Mechanistically, PD-1/PD ligand (PD-L1)-blocking antibodies stimulate T cell anti-cancer activities by removing cancer-induced brakes on the immune system (3). Indeed, accumulating evidence demonstrates the clinical significance of PD-1/PD-L1 blockade in combating cancers that are refractory to other therapeutic modalities (4). Therefore, immunotherapies based on PD-1/PD-L1 blockade will likely transform standard practices used to combat cancer and provide hope to many patients afflicted with incurable malignancies.

Owing to the growing use of ICIs in oncology, a significant number of patients receiving PD-1/PD-L1 blockade therapies experience immune-related adverse events (irAEs) affecting various organs (e.g., lung, pancreas, skin, liver, gastrointestinal tract) and endocrine and renal organ systems (5). These irAEs are generally thought to result from non-specific upregulation of immune pathways that subsequently damage normal tissues. Despite improved cancer outcomes associated with ICIs therapies, irAEs triggered by these treatments have been linked to irreversible organ damage, which has proved fatal in some cases (6). As a consequence, careful monitoring of patients receiving ICIs treatments is of great importance to protect vulnerable populations from severe irAEs. Due to the current lack of understanding of mechanisms underlying ICIs-related irAEs (1), increased awareness of ICIs-induced irAEs is needed in order to improve clinical outcomes of patients who receive ICI-based anti-cancer treatments.

Recently, a series of clinical studies have demonstrated high active tuberculosis (TB) disease rates in patients treated with PD-1/PD-L1 blockade therapies, thus suggesting that PD-1/PD-L1 blockade therapy may lead to active TB disease (7–9). Mechanistically, anti-TB effects have been associated with PD-1 function., as demonstrated using a mouse model of TB based on PD-1-deficient mice, which exhibited greater susceptibility to infection with virulent tubercle bacilli and earlier infection-induced death as compared to corresponding indicators for T-cell deficient mice (10). Nevertheless, although anti-cancer immunotherapy is a highly active clinical research area, only limited knowledge exists with regard to incidence rates of active TB disease in cancer patients receiving PD-1/PD-L1 blockade therapies. To address this concern, here we conducted a systematic review to determine TB incidence in this patient group and determined the TB-associated mortality rate for this group of patients.

## Materials and Method

### Search Strategy

Eight medical databases, including PubMed, Cochrane Library, Excerpt Medica Database (EMBASE), ClinicalTrials.gov, Chinese BioMedical Literature Database (CBM), China National Knowledge Infrastructure Database (CNKI), and Wanfang China Science and Technology Journal Database were searched for relevant publications using the following keywords (1): “anti-PD1/PD-L1 therapy”, “anti-PD1/PD-L1 immunotherapy”, “PD1/PD-L1 inhibitor”, “PD1/PD-L1 checkpoint inhibition”, “Anti-PD1/PD-L1”, “Anti-PD1 and Anti-PD-L1”, “checkpoint inhibitors”, “PD1/PD-L1 antibodies”, “PD1/PD-L1 blockade” (2); “TB” or “tuberculosis”. The search strategy used was as follows: (1) AND (2). No limitation was imposed with regard to study characteristics of region or study participant race or age during the search. Language was mainly limited to Chinese and English. Only available data from published articles were collected; data from unpublished papers were not included.

### Inclusion Criteria

Inclusion criteria were as follows: (1) randomised controlled trials, nonrandomised controlled trials, case-control studies, cohort studies, cross-sectional studies, retrospective studies, and case reports on clinical characteristics of TB activation after administration of ICIs; (2) studies involving patients treated with ICIs, both single-agent therapy and ICIs used in combination with other agents. Exclusion criteria included the following: (1) articles published repeatedly; (2) studies lacking research indicators required for meta-analysis.

### Data Extraction

Two investigators performed study selection independently. Investigators first screened the literature by reviewing literature abstracts to exclude articles that obviously did not meet study inclusion criteria. Next, investigators rescreened articles retained after abstract review by reading the full text of each article. If any disagreement arose regarding selection of literature papers, a third evaluator was consulted to render a final decision on whether to include or exclude the article in question.

### Statistical Analysis

The TB rate in the group of patients who received PD-1/PD-L1 blockade therapy was assessed then a meta-analysis was conducted during which odds ratios (ORs) and 95% CIs values were calculated using the inverse variance-weighted average statistical method. Heterogeneity was assessed using chi-square and I^2^ statistical tests. A p value of ≥0.10 together with an I^2^ value of ≥50% indicated significant heterogeneity, while an I^2^ value of ≤50% indicated acceptable inter-study heterogeneity. If significant inter-study heterogeneity was detected, data were analysed using the fixed-effects model; otherwise, the random-effects model was selected. Publication bias was detected using a funnel plot. Sensitivity analysis was performed to explore effects of trial bias risk on outcomes. Sensitivity analysis involved comparing results of analysis of all studies followed by analysis of remaining studies after one study at a time was removed, with results of this analysis showing whether removal of any single study significantly impacted overall results. Finally, results of meta-analysis results were presented as forest plots.

Meta-analysis was performed using RevMan version 5.3 (Cochrane Collaboration), with p<0.05 considered statistically significant. In order to reduce the influence of heterogeneity between the included studies on final conclusion, the random effects model was used for meta‐analysis.

## Results

**Figure 1:**
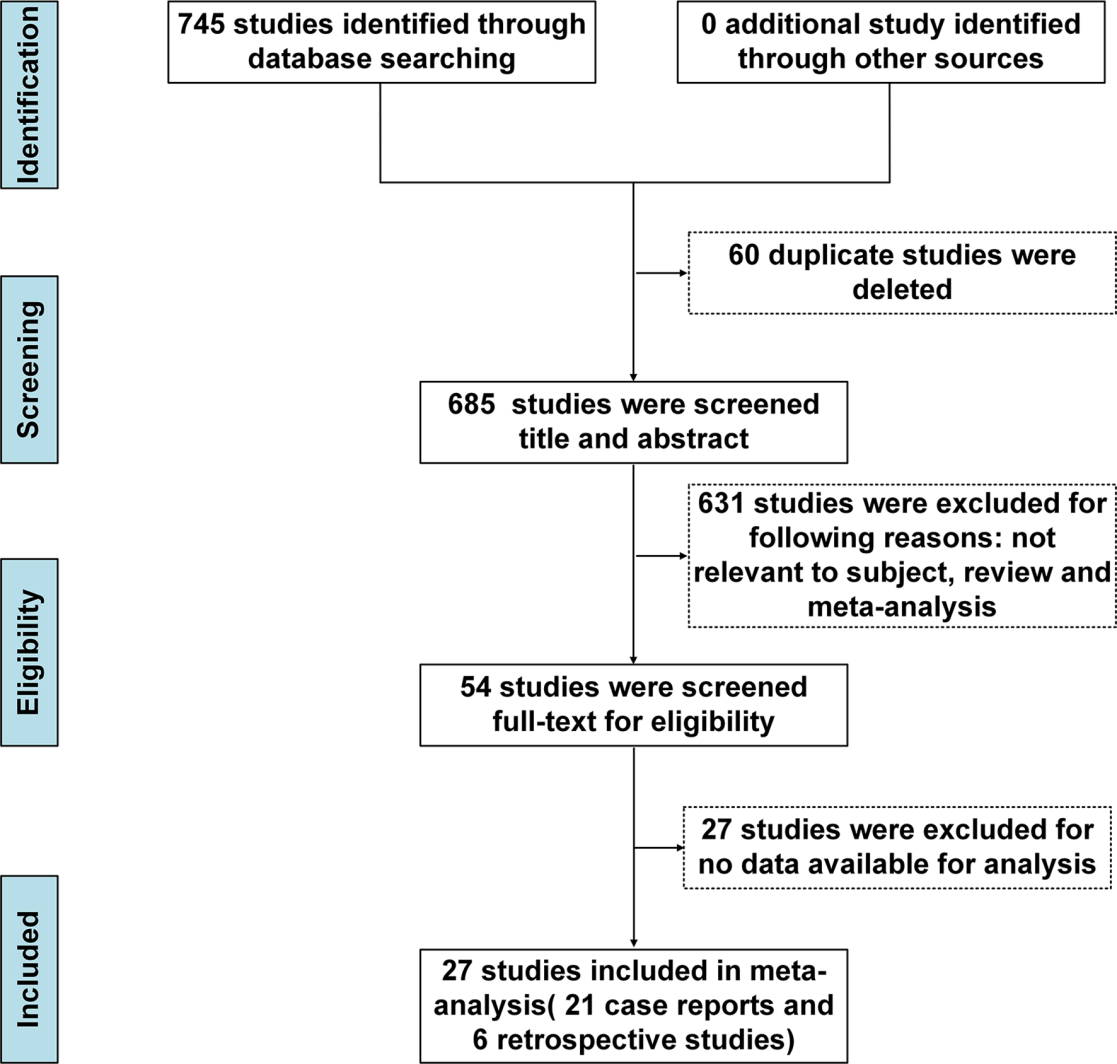
PRISMA flow chart of inclusion process. PRISMA, Preferred Reporting Items for Systematic Reviews and Meta-Analyses.

We initially identified 745 records after implementing the search strategy. After elimination of duplicates, 685 records remained. Subsequent screening of titles and abstracts of the remaining records led to exclusion of 631 irrelevant records, leaving 54 articles. These articles were re-evaluated based on full-text contents, resulting in exclusion of 27 articles based on incomplete data. Ultimately, 27 studies met the inclusion criteria and were included in our meta-analysis (**Figure 1**), with the reference list of all included studies summarised in **Table 1**. Of these 27 studies, 21 were case reports and 6 were retrospective studies. All selected studies were published after 2016.

**Table 1:** Baseline characteristics of patients included in our analysis.

Study	Age, year	Sex	Region or country of origin of report	Cancer	Therapy before PD-1/PD-L1			Use of steroids before diagnosis of TB	PD-1/PD-L1
					surgery	radiotherapy	chemotherapy		
Van Eeden 2019 (11)	56	Female	South Africa	NSCLC	No	Yes	Yes	No	Nivolumab
He 2018 (12)	65	Female	China	Melanoma	Yes	No	Yes	No	Pembrolizumab
Fujita 2016 (13)	72	Male	Japan	NSCLC	No	No	Yes	No	Nivolumab
Viatgé 2020 (14)	49	Male	France	Melanoma	No	No	No	No	Nivolumab
Fujita 2020 (1) (15)	79	Female	Japan	NSCLC	No	No	Yes	No	Nivolumab
Fujita 2020 (2) (15)	74	Male	Japan	NSCLC	No	Yes	Yes	No	Pembrolizumab
Fujita 2020 (3) (15)	79	Male	Japan	NSCLC	No	No	Yes	No	Nivolumab
Fujita 2020(4) (15)	64	Male	Japan	NSCLC	No	No	Yes	No	Pembrolizumab
Fujita 2020(5) (15)	77	Female	Japan	NSCLC	No	Yes	Yes	No	Durvalumab
Song 2020 (16)	69	Male	Canada	Nasopharyngeal carcinoma	No	No	No	No	Avelumab
Sirgiovanni 2021 (17)	44	Male	Germany	SCLC	No	Yes	Yes	No	Nivolumab,Ipilimumab
Kato 2020 (18)	75	Female	Japan	NSCLC	No	Yes	Yes	No	Durvalumab
Anastasopoulou 2019(1) (19)	76	Female	Greece	Melanoma	Yes	No	Yes	Yes	Nivolumab+/-Ipilimumab^a^
Anastasopoulou 2019(2) (19)	85	Male	Greece	Melanoma	Yes	No	Yes	No	Atezolizumab
Murakami 2020 (20)	73	Male	Japan	NSCLC	No	No	No	Yes	Pembrolizumab
Inthasot 2019 (21)	69	Male	Belgium	Lung adenocarcinoma	No	No	Yes	No	Nivolumab
Barber 2019(1) (22)	59	Male	USA	Nasopharyngeal carcinoma	No	No	Yes	No	Nivolumab
Barber 2019(2) (22)	83	Male	USA	MCC	No	No	No	No	Pembrolizumab
Suliman 2020 (23)	58	Female	Qatar	Lung adenocarcinoma	No	No	No	No	Pembrolizumab
Papadaki 2020 (24)	62	Male	Greece	Lung adenocarcinoma	No	Yes	Yes	No	Durvalumab
Jensen 2018 (25)	56	Male	Denmark	NSCLC	No	Yes	Yes	No	Nivolumab
Chu 2017 (26)	59	Male	China	Lung adenocarcinoma	No	No	Yes	No	Nivolumab
Picchi 2017(1) (27)	50	Male	France	Melanoma	NA	NA	NA	No	Pembrolizumab
Picchi 2017(2) (27)	64	Male	France	NSCLC	NA	NA	NA	No	Nivolumab
Takata 2018 (28)	75	Male	Japan	Lung adenocarcinoma	No	Yes	Yes	No	Nivolumab
Tsai 2019 (29)	49	Male	Taiwan	SCC of hard palate	No	Yes	Yes	No	Nivolumab
Lee 2016 (30)	87	Male	China	Hodgkin’s Lymphoma	No	Yes	Yes	Yes	Pembrolizumab
Elkington 2018 (31)	62	Female	UK	Melanoma	Yes	No	No	No	Ipilimumab,Pembrolizumab
Tetikkurt 2018 (32)	53	Male	Turkey	SCC of hard palate and maxilla	Yes	Yes	Yes	No	Pembrolizumab
Im 2019(1) (7)	63	Male	South Korea	Lung adenocarcinoma	NA	NA	Yes	No	Nivolumab
Im 2019(2) (7)	79	Male	South Korea	SCC of lung	NA	NA	Yes	Yes	Pembrolizumab
Im 2019(3) (7)	59	Female	South Korea	Lung adenocarcinoma	NA	NA	Yes	Yes	Nivolumab
Byeon 2020(1) (33)	57	Female	South Korea	NSCLC	NA	NA	NA	No	Nivolumab
Byeon 2020(2) (33)	61	Male	South Korea	NSCLC	NA	NA	NA	No	Nivolumab
Byeon 2020(3) (33)	84	Female	South Korea	NSCLC	NA	NA	NA	No	Pembrolizumab

SCC, squamous cell carcinoma; NSCLC, non-small-cell lung cancer; SCLC, small-cell lung cancer; MCC, Merkel cell carcinoma; TB, tuberculosis; NA, not available.

a The patient enrolled in a clinical trial (ClinicalTrials.gov ID: NCT03068455) and was randomized to receive monotherapy with nivolumab 240 mg every 2 weeks versus the combination of nivolumab with ipilimumab 1 mg/kg every 3 weeks.

### Demographic and Clinical Characteristics of TB Patients

A total of 35 TB cases were detected in the group of patients receiving PD-1/PD-L1 blockade therapies, with patient demographic and clinical characteristics summarised in **Table 1**. The median age of TB patients within the treatment group was 64 years (range: 44-87 years). The majority of individuals were male, accounting for 68.6% (24/35) of all TB patients included in our analysis. Of the 35 TB cases, 23 (65.7%) were diagnosed with lung cancer, 6 (17.1%) with melanoma, 2 (5.7%) with nasopharyngeal carcinoma, and 4 (11.4%) with other types of cancer. Nivolumab (51.4%) was the most frequently used anti-cancer PD-1/PD-L1 blockade therapy, followed by pembrolizumab (34.3%) and durvalumab (8.6%). PD-1/PD-L1 treatment duration was 22 to 730 days (median duration of 120 days) before clinical symptoms of active TB were detected. Notably, pulmonary TB was the most common form of TB observed, with an incidence rate of 77.1% (27/35) of cases. Meanwhile, 17.1% (6/35) of cases were diagnosed with extrapulmonary TB and 5.7% (2/35) of cases were diagnosed with concurrent extrapulmonary and pulmonary TB (**Table 2**). For the 35 TB patients, clinical outcomes were recorded in 20 patients, whereby 14 cases (70%) were cured or experienced remission and 6 cases (30%) died from active TB disease (**Table 3**).

**Table 2:** Detailed clinical data of TB patients included in this study.

Study	TB-affected organ	Radiology	TST	IGRA	AFB	PCR	Culture	Pathology	Time interval between initiation of PD-1/PD-L1 therapy and diagnosis of TB (day)
Van Eeden 2019 (11)	Lung	+			+^b^				180
He 2018 (12)	Lung	+					+^d^	+	240
Fujita 2016 (13)	Lung	+		+		+^d^	+^d^	–	120
Viatgé 2020 (14)	Lung	+	+						90
Fujita 2020(1) (15)	Knee joint	NA							80
Fujita 2020(2) (15)	Lung	NA							135
Fujita 2020(3) (15)	Lung	NA							29
Fujita 2020(4) (15)	Lung	NA							22
Fujita 2020(5) (15)	Cervical and hilar lymph nodes	NA							398
Song 2020 (16)	Laryngeal	+			+^b^				NA
Sirgiovanni 2021 (17)	Lung	+			+^b^	+^d^			NA
Kato 2020 (18)	Lung	+		+	+^b^	+^b^			NA
Anastasopoulou 2019(1) (19)	Lung	+				+^d^			210
Anastasopoulou 2019(2) (19)	Lung	NA					+^b^		210
Murakami 2020 (20)	Lung	+			+^b^	+^b^	+^b^		548
Inthasot 2019 (21)	Lung	+				+^d^	+^d^		NA
Barber 2019(1) (22)	Lung and rectum	+			+^bc^	+^b^	+^dg^	+	45
Barber 2019(2) (22)	Lung	+		+			+^e^	+	240
Suliman 2020 (23)	Lung	+			+^bd^	+^d^			NA
Papadaki 2020 (24)	Lung	+					+^d^		660
Jensen 2018 (25)	Lung	+				+^e^		+	NA
Chu 2017 (26)	Pericardium	NA					+^h^	+	45
Picchi 2017(1) (27)	Pleura	+	+					+	90
Picchi 2017(2) (27)	Bone					+^f^	+^f^	+	30
Takata 2018 (28)	Lung	+			+^b^	+^b^	+^b^		NA
Tsai 2019 (29)	Lung	+			+^b^	+^b^	+^b^		90
Lee 2016 (30)	Lung	NA					+^b^		NA
Elkington 2018 (31)	Lung and liver	+					+^d^	+	730
Tetikkurt 2018 (32)	Lung	NA					+^NA^		NA
Im 2019(1) (7)	Lung	+					+^d^		630
Im 2019(2) (7)	Lung	+				+^b^			240
Im 2019(3) (7)	Lung	+					+^b^		30
Byeon 2020(1) (33)	Lung	NA							120
Byeon 2020(2) (33)	Lung	+					+^d^		NA
Byeon 2020(3) (33)	Lung	NA							30

TB, tuberculosis; BALF, bronchoalveolar lavage fluid; NA, not available; +, indicates positive.

^b^, sputum; ^c^, rectum sample; ^d^, BALF; ^e^, lung; ^f^, bone; ^g^, stool; ^h^, pericardial effusion.

**Table 3:** Treatment outcomes of TB patients with PD-1/PD-L1 therapy.

Study	Regimen of anti-TB treatment		Outcome of TB treatment	Interruption of PD-1/PD-L1 therapy	Reinitiation of PD-1/PD-L1 therapy	Tumor response to PD-1/PD-L1 therapy
Van Eeden 2019 (11)	HRZE	Early improvement		Yes	Yes	Response initially
He 2018 (12)	HRZE-HRZ-SEMfx	Early improvement		Yes	Yes	CR
Fujita 2016 (13)	NA	NA		Yes		Response initially
Viatgé 2020 (14)	HRZE	Early improvement		No		Response initially
Fujita 2020(1) (15)	HRZE	NA		NA		NA
Fujita 2020(2) (15)	HRZE	NA		NA		NA
Fujita 2020(3) (15)	HRZE	NA		NA		NA
Fujita 2020(4) (15)	HRZE	NA		NA		NA
Fujita 2020(5) (15)	HRZE	NA		NA		NA
Song 2020 (16)	HRZE	Early improvement		Yes		NA
Sirgiovanni 2021 (17)	HRZE	Early improvement		Yes		Response initially
Kato 2020 (18)	NA	Early improvement		Yes		Response initially
Anastasopoulou 2019(1) (19)	HRZE	Died		Yes		NA
Anastasopoulou 2019 (2) (19)	HRZ	NA		No		NA
Murakami 2020 (20)	HRZE	Cured		Yes	Yes	PR
Inthasot 2019 (21)	NA	NA		NA		Response initially
Barber 2019(1) (22)	HRZE-SRLzdMfx	Died		Yes		Response initially
Barber 2019(2) (22)	HRZE-HRfb-LfxRfb	Cured		Yes	Yes	PR
Suliman 2020 (23)	HRZE	Early improvement		Yes		PD
Papadaki 2020 (24)	NA	Early improvement		Yes		Response initially
Jensen 2018 (25)	NA	NA		Yes		Response initially
Chu 2017 (26)	NA	Cured		Yes	Yes	PR
Picchi 2017(1) (27)	NA	Cured		No		NA
Picchi 2017(2) (27)	NA	Died		Yes		NA
Takata 2018 (28)	HRZE-HEMfx-HRZE/HR	Cured		Yes	Yes	PR
Tsai 2019 (29)	NA	Died		Yes		NA
Lee 2016 (30)	HRE	NA		Yes		PR
Elkington 2018 (31)	NA	NA		NA		SD
Tetikkurt 2018 (32)	NA	NA		Yes	Yes	CR
Im 2019(1) (7)	HRZE	DRESS syndrome developed after initiating anti-TB medication.		NA		PR
Im 2019(2) (7)	HRZE	Died		NA		SD
Im 2019(3) (7)	HRZE	Died		NA		PD
Byeon 2020(1) (33)	NA	NA		NA		PD
Byeon 2020(2) (33)	NA	NA		No		PR
Byeon 2020(3) (33)	NA	NA		No		PD

TB, tuberculosis; H, isoniazid; R, rifampin; Z, pyrazinamide; E, ethambutol; S, streptomycin; Mfx, moxifloxacin; Lzd, linezolid; Rfb, rifabutin; Lfx, levofloxacin.

CR, complete response; PR, partial response; SD, stable disease; PD, progressive disease; NA, not available.

### TB Risk Associated With PD-1/PD-L1 Blockade Therapy

**Figure 2:**
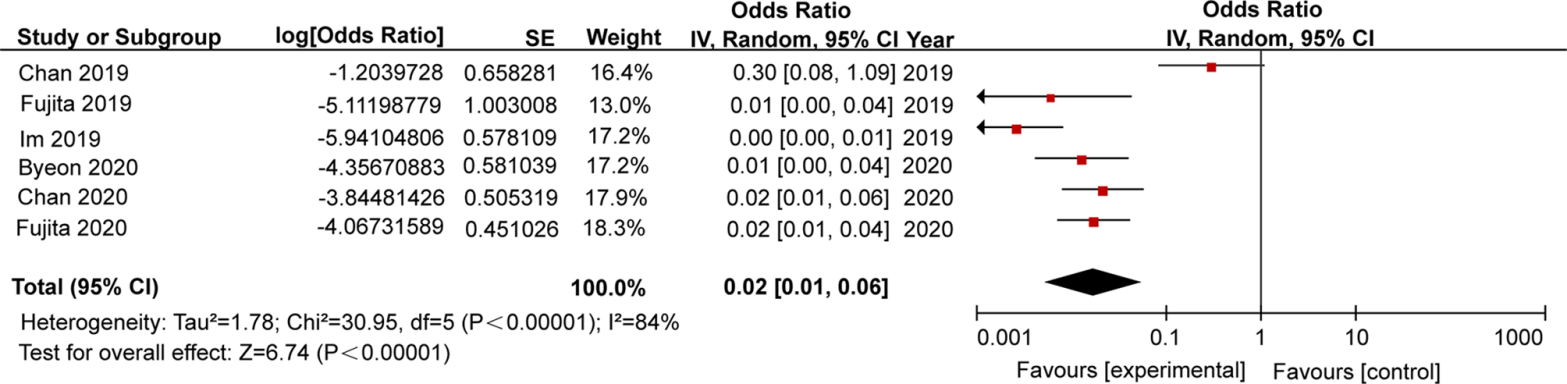
Incidence of tuberculosis among patients treated with PD-1/PD-L1 blockade.

We further analysed pooled data in order to determine TB rates in patients who had received PD-1/PD-L1 blockade therapies. Six retrospective studies were included for meta-analysis, including two studies from each of three countries (Singapore, Japan, South Korea). Of these studies, a total of 19 active TB cases were reported (**Table 4**). As shown in **Figure 2**, pooled analysis results indicated that the TB rate in this population was 2,000 cases per 100,000 persons [95% confidence interval (95% CI): 1,000-6,000 cases per 100,000 persons]. Due to the small numbers of study subjects in each of these retrospective studies, significant inter-study heterogeneity was detected during our analysis (p<0.00001, I^2^ = 84%). Nevertheless, the results for 2019 revealed that the average TB rate based on data obtained from studies conducted in Singapore, Japan, and South Korea was 55 cases (range, 10-99 cases) per 100,000 (35). Therefore, the estimated rate of association of active TB incidence with PD-1/PD-L1 blockade therapy was 35 times higher than that of the general population.

**Table 4:** Characteristics of study population in the 6 retrospective studies with prediction models for TB incidence.

Study	Country	Study type	Period	Cancer	Patients	TB activate after PD-1/PD-L1 therapy
Chan 2019 (34)	Singapore	Retrospective study	2014-2019	NSCLC	13	3
Fujita 2019 (8)	Japan	Retrospective study	2015-2019	NSCLC	167	1
Im 2019 (7)	South Korea	Retrospective study	2014-2018	Solid cancer^i^	1144	3
Byeon 2020 (33)	South Korea	Retrospective study	2015-2018	NSCLC	237	3
Chan 2020 (9)	Singapore	Retrospective study	2014-2019	NSCLC	191	4
Fujita 2020 (15)	Japan	Retrospective study	2016-2019	NSCLC	297	5

NSCLC, non-small-cell lung cancer; ^i^, including lung cancer, melanoma, lymphoma, gastric cancer, head and neck cancer, thymic cancer, mesothelioma and sarcoma.

### Publication Bias Assessment

**Figure 3:**
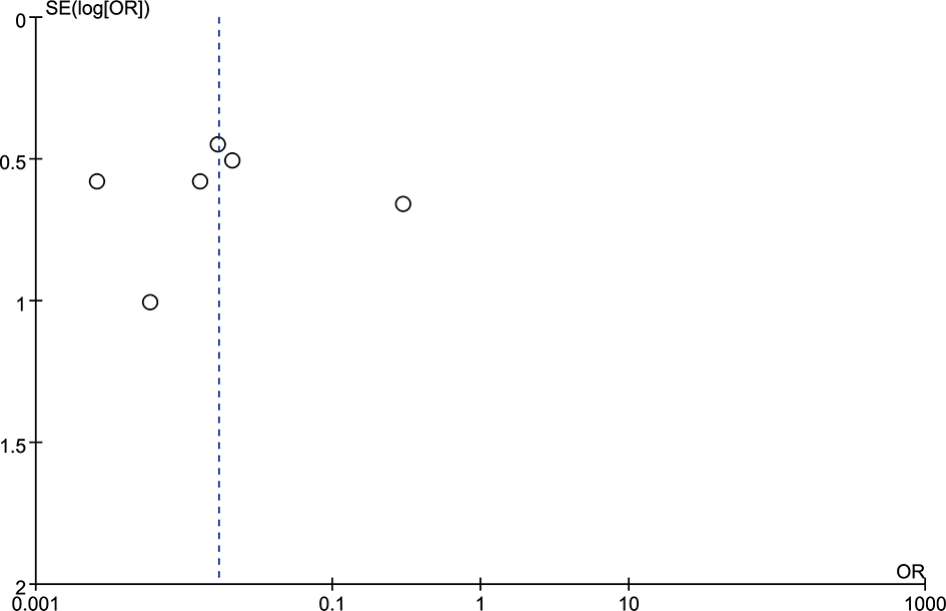
The funnel plot indicated a trend in publication bias. The dotted line is provided as a reference to the composite estimate from the combined studies.

A funnel plot was constructed to evaluate publication bias, which indicated no evidence of publication bias (**Figure 3**). Judging from the graph, the funnel plot was roughly symmetrical, thus indicating that no publication bias was detected in this meta-analysis.

### Sensitivity Analysis

**Figure 4:**
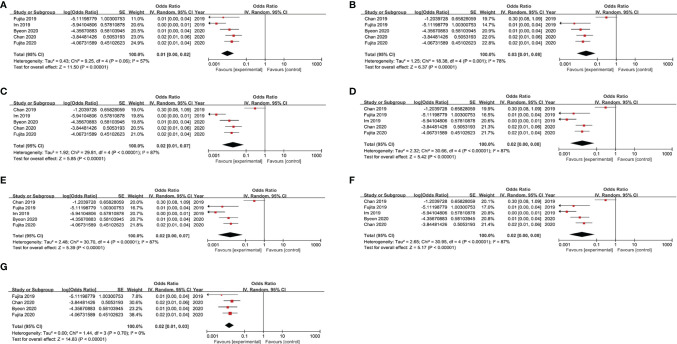
Results of sensitivity analysis after removing each study in turn. **(A)** Sensitivity analysis by removing Chan 2019; **(B)** Sensitivity analysis by removing Im 2019. **(C)** Sensitivity analysis by removing Fujita 2019. **(D)** Sensitivity analysis by removing Byeon 2020. **(E)** Sensitivity analysis by removing Chan 2020. **(F)** Sensitivity analysis by removing Fujita 2020. **(G)** Sensitivity analysis by removing the Chan 2019 and Im 2019.

Due to the fact that a high level of inter-study heterogeneity was detected (p<0.00001, I^2^ = 84%), we carried out sensitivity analysis by removing a single study at a time and analysing results obtained for the remaining studies to determine if the omitted study had introduced excessive heterogeneity into the overall results (**Figure 4**). We found that the level of heterogeneity among studies was reduced when Chan 2019 or Im 2019 were removed, as reflected by changes in overall odds ratios after removal of each of these studies (**Figures 4A, B**). After additional sensitivity analysis was performed, the Chan 2019 and Im 2019 articles were both removed from the meta-analysis (**Figure 4G**). After removing the two studies, no significant heterogeneity was detected across studies (p = 0.7, I^2^ = 0%), and the overall odds ratio was consistent with that obtained from our initial meta-analysis that included the two publications in question (**Figure 2**).

## Discussion

The introduction of ICIs (PD-1 and PD-L1 inhibitors) provides new hope for patients afflicted with cancer (36). However, increasing evidence suggests a significantly high correlation between PD-1/PD-L1 blockade therapy administration to cancer patients and active TB disease incidence, thus prompting us to conduct this meta-analysis to assess TB risk in patients treated with PD-1/PD-L1 inhibitors. Our data demonstrated that the TB rate in patients receiving PD-1/PD-L1 blockade therapies was 2,000 cases per 100,000, a rate that was 35 times higher than that of the general population. This result suggested that administration of PD-1/PD-L1 blockade therapies to patients interfered with immunological control of latent TB infections that led to increased activity of tubercle bacilli and active TB disease. This result aligns with previous histopathological data demonstrating that immune system control over latent TB disease caused by *Mycobacterial tuberculosis* (MTB) infection must be carefully balanced against sufficient immune cell activation-based inhibition of pathogen growth in order to avoid excessive inflammation and associated pathological damage (37). In fact, a recent experimental study by Tezera and colleagues revealed that hypoxia within TB lesions led to up-regulated expression of PD-1/PD-L1 axis proteins (38). Meanwhile, results of another study focusing on PD-1/PD-L1 blockade immunotherapy indicated that inhibition of this pathway could stimulate multiple types of immune cells within granulomas (e.g., T cells, macrophages, dendritic cells), thereby resulting in granuloma collapse and excessive immunopathology (39). These results are consistent with results of studies of MTB-infected patients and of human 3D cell culture-based TB models that suggest that PD-1 inhibition accelerates MTB growth *via* excessive TNF-α secretion (38). Taken together, these results highlight the important role of PD-1 in fine-tuning the balance between pro- and anti-inflammatory responses against tubercle bacilli to avoid tissue damage.

Another notable finding of our analysis pertains to the extremely high TB-associated mortality rate in the group of patients receiving PD-1/PD-L1 blockade therapy. Several possible factors may explain this association. For one, developing tumours must escape attack from the host immune system. In view of the fact that tumour cells can uniquely acquire energy from glycolysis rather than from mitochondrial oxidative phosphorylation as a mechanism for evading immune attack, the fundamental immunodeficient state of malignancies that is associated with this survival mechanism can lead to worse clinical outcomes in patients afflicted with both cancer and TB. In addition, malnutrition, which is commonly associated with cancer, may be further aggravated by TB disease to account for excessively high mortality in patients afflicted with both cancer and TB. Taken together, our data indicate that more attention should be paid to managing TB and cancer in patients afflicted with both diseases in order to improve clinical outcomes.

Importantly, the time to onset of active TB during administration of PD-1/PD-L1 blockade therapy provides an important clue for use in formulating an appropriate follow-up strategy. Results of our analysis revealed that the reported median time to onset of active TB after starting PD-1/PD-L1 blockade therapy was 120 days, a time period that was slightly longer than that observed in patients receiving anti-TNF-α treatment (~12 weeks) (40). In view of the potential role of PD-1/PD-L1 blockade therapy in supporting TB emergence by inducing excessive secretion of the proinflammatory cytokine TNF-α, TNF-α appears to be a double-edged sword such that it promotes TB disease progression due to unbalanced expression of TNF-α that may alter host-pathogen interactions within granulomas. Thus, our data suggest that screening for latent TB disease prior to immunotherapy should be conducted not only in patients who are starting anti-TNF therapy, but also in patients who will receive PD-1/PD-L1 blockade therapy. For patients with latent TB disease, routine follow-up testing during immunotherapy should be conducted to monitor TB disease status and detect TB reactivation early. Nevertheless, the prolonged interval between onset of TB after initiation of anti-PD-1/PD-L1 treatment as compared to that observed after initiation of anti-TNF-α therapy indicates that TNF-α acts downstream the point of PD-1/PD-L1 blockade to trigger reactivation of latent TB.

We also acknowledge several obvious limitations of this study. First, several important non-English and non-Chinese studies reporting recurrence rates of TB among patients treated with PD-1/PD-L1 blockade might have been omitted from in our review. Second, due to the high cost of PD-1/PD-L1 blockade therapy, all descriptive studies were obtained from developed counties with low TB burden, leading to biased estimates of TB risk due to differences in availability of anti-PD-1/PD-L1 therapy. Third, in view of the small numbers of TB patients in these studies, none of the studies used in our final analysis provided enough information to delineate risk factors associated with TB reactivation in patients receiving anti-PD-1/PD-L1 therapy. Fourth, the interval between immunotherapy and TB reactivation was calculated according to self-reported clinical symptoms and thus may have led to the underestimation of the TB incidence rate in this population. Fifth, the lack of a consistent case definition of TB across all studies may complicate interpretation of our findings. Sixth, although comorbidities are important factors that can impact rates of active TB disease and morbidity, complete information on patient comorbidities (e.g., HIV, diabetes, kidney disease) was not provided in these papers for inclusion in our analysis. The comorbidities may put patients at high risk of TB infection and TB morbidity. Meanwhile, patients with certain types of cancer (e.g., head and neck carcinomas) are at especially high risk of contracting active TB, with risk of TB morbidity in these patients reported to be 2.86 to 16 times that found in the general population (41, 42). However, careful review of the studies that were included in our meta-analysis revealed only one study that included head and neck carcinoma patients (35/1144, 3.1%). Therefore, due to the fact that only a small proportion of patients in that study were afflicted with this type of cancer, the impact of the results of that study on our final conclusion was small. Finally, different types of anti-TB regimens may affect TB mortality rates. Nevertheless, due to the complications stemming from TB treatments (e.g., drug tolerance, decreased *in vitro* drug susceptibility) and high treatment costs, significant heterogeneity in mortality rates was noted across studies that thus precluded us from analysing the effect of anti-TB therapeutic regimens on TB-associated mortality rates in these TB patients, warranting further study.

Due to the fact that a high level of inter-study heterogeneity was detected, we carried out sensitivity analysis that revealed that two studies, Chan 2019 and Im 2019, accounted for most of the observed heterogeneity across studies. Upon further analysis, the small sample size in Chan 2019 and different tumour types studied in Im 2019 may have been largely responsible for increased heterogeneity. Importantly, after these studies were omitted, sensitivity analysis results were consistent with our previous meta-analysis results.

In conclusion, our results demonstrate that clinical use of PD-1/PD-L1 inhibitors significantly increases TB reactivation risk. In addition, we observed an extremely high mortality rate due to active TB disease in patients who received PD-1/PD-L1 blockade therapy. Thus, routine follow-up monitoring of latent TB in patients receiving ICIs-based immunotherapies is of great importance for detecting TB reactivation and improving disease prognosis. Ultimately, more research is required to better understand the relative contribution of each risk factor to the increased incidence of TB reactivation in this patient population.

## Data Availability Statement

The original contributions presented in the study are included in the article/supplementary material. Further inquiries can be directed to the corresponding authors.

## Author Contributions

Concept and design: YP and MG. Acquisition, analysis, or interpretation of data: KL, DW, and CY. Drafting of the manuscript: YP, MG, and KL. Critical revision of the manuscript for important intellectual content: all authors. Statistical analysis: MQ, QL, WR, and SL. Administrative, technical, or material support: all authors. All authors critically reviewed and approved the final manuscript.

## Funding

This work was supported by the Beijing Hospitals Authority Ascent Plan (DFL20191601), the Beijing Hospitals Authority Clinical Medicine Development of Special Funding (ZYLX202122), and the Capital Clinical Diagnosis and Treatment Technology Research and Demonstration Application (Z191100006619077).

## Conflict of Interest

The authors declare that the research was conducted in the absence of any commercial or financial relationships that could be construed as a potential conflict of interest.

## Publisher’s Note

All claims expressed in this article are solely those of the authors and do not necessarily represent those of their affiliated organizations, or those of the publisher, the editors and the reviewers. Any product that may be evaluated in this article, or claim that may be made by its manufacturer, is not guaranteed or endorsed by the publisher.
